# Real-Time Object Detection and Classification by UAV Equipped with SAR

**DOI:** 10.3390/s22052068

**Published:** 2022-03-07

**Authors:** Krzysztof Gromada, Barbara Siemiątkowska, Wojciech Stecz, Krystian Płochocki, Karol Woźniak

**Affiliations:** 1 Institute of Automatic Control and Robotics, Warsaw University of Technology, 02-525 Warsaw, Poland; barbara.siemiatkowska@pw.edu.pl (B.S.); krystian.plochocki@pw.edu.pl (K.P.); karol.wozniak@pw.edu.pl (K.W.); 2 Faculty of Cybernetics, Military University of Technology, 00-908 Warsaw, Poland; wojciech.stecz@wat.edu.pl

**Keywords:** computer vision, pattern recognition, synthetic aperture radar (SAR), unmanned aerial vehicles (UAV), you only look once (YOLO), deep-learning, deep neural networks (DNN)

## Abstract

The article presents real-time object detection and classification methods by unmanned aerial vehicles (UAVs) equipped with a synthetic aperture radar (SAR). Two algorithms have been extensively tested: classic image analysis and convolutional neural networks (YOLOv5). The research resulted in a new method that combines YOLOv5 with post-processing using classic image analysis. It is shown that the new system improves both the classification accuracy and the location of the identified object. The algorithms were implemented and tested on a mobile platform installed on a military-class UAV as the primary unit for online image analysis. The usage of objective low-computational complexity detection algorithms on SAR scans can reduce the size of the scans sent to the ground control station.

## 1. Introduction

Unmanned aerial vehicles are the fastest-growing segment of the military aerospace market. Their sensors’ capabilities form the critical feature of each unit type. A wide range of UAV payloads (including light electro-optical/infrared (EO/IR) systems, synthetic aperture radars (SAR) [1], SIGINT (signal intelligence), and EW (electronic warfare)) is systematically enriched and introduced to the military market. This technological trend is connected with civilian demand to use unmanned aerial systems in search-and-rescue missions or environmental protection tasks.

Presently, UAVs of the MALE (medium-altitude long-endurance) class can carry out ISR (intelligence, surveillance, and reconnaissance) missions and provide the needed data. Various sensors have become the heart of weapons systems. Due to the rising popularity of MALE systems, UAVs belonging to smaller classes are being equipped with progressively more advanced intelligence systems, i.e., payload and algorithms of pattern recognition and data fusion. An excellent example of such a system is a SAR system used for vessel-type recognition [2]. Another important application of SAR radars is UAV navigation support in all weather conditions [3,4,5].

The systems used in the military or rescue services to identify dangerous objects must have a correctly planned flight trajectory in the neighborhood of the recognized object [4]. The SAR will generate a valid scan only if it can collect data from a given area at a length that is at least equal to the length of the synthetic aperture $L_{A}$ , determined by the given parameters: the resolution and slant range. The procedure for determining the length of a synthetic aperture is presented in [4,5].

**Figure 1:**
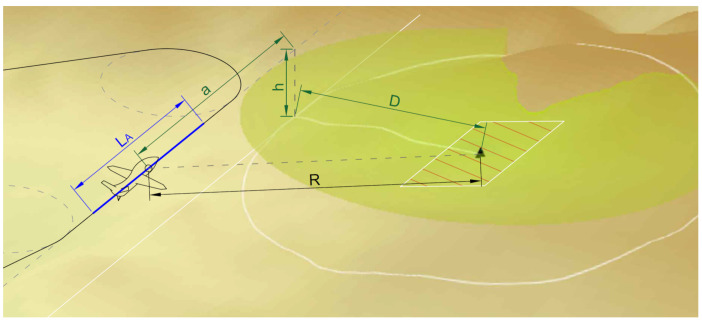
Graphical representation of the SAR scan’s geometrical parameters: R—slant range, D—ground distance, h—flight height (above ground level), a—offset, $L_{A}$ —synthetic aperture length required of flight distance. The scanned area is marked on the map with a polygon.

The operating principle of the radar and the corresponding geometrical variables are shown in Figure 1. In military applications, the analysts define the resolution at which SAR scans should be performed using the NIIRS (National Imagery Interpretability Rating Scale) [6]. It can be assumed that SAR radars can provide imagery from Level 1 (enabled to distinguish major land uses) to Level 6 (can identify car body styles) in the NIIRS rating.

**Figure 2:**
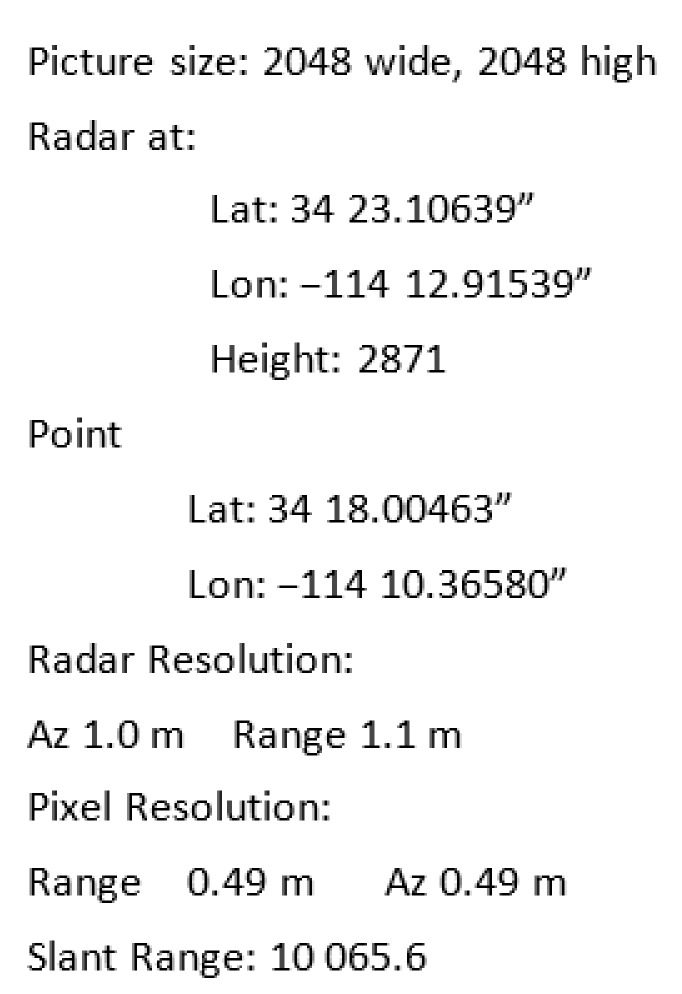
Sample metadata of the SAR’s scan. Pixel resolution is presented.

In order to recognize the object efficiently, information about the so-called pixel resolution from the attached metadata is needed. Depending on the resolution, one can determine how many pixels a vehicle or a building can occupy in the scan. In the tests, the GDAL [7] package was used to read the metadata of scans. A fragment of sample metadata is shown in Figure 2.

For each UAV platform with SAR installed, the optimal scanning parameters under various conditions are determined based on the data provided by the analyst. These parameters can be translated directly into the value of the synthetic aperture. Given the value of the synthetic aperture, the analyst can plan the flight trajectory in the neighborhood of the recognized object to ensure a flight along the straightest possible line. The SAR is equipped with a MoCo (motion compensation) subsystem, but it can only reduce UAV vibration to a certain extent. Therefore, in the planning process, it is crucial to consider the weather conditions at the location of the recognized object. Currently, intensive work is underway to construct online weather forecasts for unmanned systems flying up to several thousand meters above ground level. It can be assumed that in the coming years, the mechanisms of automatic planning and the correction of routes will become standard equipment of the ground control station.

The primary purpose of our research is to build an algorithm that will detect specific objects in SAR images in real-time. The processing of high-resolution images is computationally demanding, especially with the limitations of mobile hardware (both processing power and RAM memory). Hence, selecting suitable methods is critical. This article presents the methods of recognizing objects of various sizes on SAR scans made by UAVs.

The results of the algorithms embedded on the Jetson Tegra TX2i mobile platform are presented. Both the central processing unit (CPU) and the graphics processing unit (GPU) are used for calculations. The performance of each step of the tested algorithms is discussed in detail. Premises when the algorithms will benefit from redirection of calculations to the graphics processor and the CUDA library are indicated. The tested UAV performed several processes simultaneously on the mission management computer (Jetson platform). In this context, transferring the computation related to image recognition to the GPU is justified, even when processing times are longer. The efficiency of the Jetson processing units also prompts this.

A unique role in the presented research is played by analyzing the presented algorithms’ effectiveness on mobile computing platforms that can be used on UAVs. The use of mobile platforms is promising for several reasons:

1. Only such platforms can feasibly be installed on an UAV;
2. Mobile computing platforms are becoming more and more efficient;
3. Some of these platforms meet the requirements imposed on computers used in industry and aviation.

It should also be remembered that only industrial computers of the class similar to the Tegra TX2i can be certified for use in airborne platforms (in line with aviation standards). As the certification of computers onboard the aerial platform is now mandatory, benchmarking the performance of these platforms is essential and will become more frequent.

The article is organized as follows: in Section 2, the related methods have been presented. Section 3 describes the classical vision system used in the research. Section 4 presents the results of classifying a selected class of objects (tanks) using YOLOv5 networks. Section 5 presents a new method that involves the integration of YOLO with classical image-processing methods. Section 7 presents the conclusions and future work.

## 2. Related Methods

Automatic SAR image analysis and target recognition became one of the research hotspots for remote sensing technology [8]. These images are usually affected by speckle noise [9] and distortion effects (such as shadowing). In addition, they contain high local contrasts in intensity. These features make SAR images large, noisy, and difficult to interpret.

It should be mentioned that there are advanced image-processing algorithms that are SAR-specific. Usually, they are based on the fact that SAR imagery is obtained as a complex value (which means that it consists of amplitude and phase information). For example, interferometry, or more advanced CCD (coherent change-detection) algorithms [10] can be used to detect sub-wavelength size changes in the images. In contrast to the described algorithms, they require at least two scans from the same position to work. Due to higher turbulence levels in lightweight vehicles, flying the same route with enough precision to perform these analyses is usually impossible. Therefore, this article focuses on other forms of image processing and object identification. The CCD or interferometry algorithms could be used to add additional contextual information to the images (see Section 3).

We can divide SAR amplitude imagery analysis methods into two primary groups: classical processing, and classification using convolutional networks (CNNs). The classic automated computer-aided classification system implements four main steps: pre-processing, segmentation, feature extraction, and classification [11].

The main task of pre-processing is to reduce the noise [12]. Usually, filters (linear or nonlinear) or wavelet transforms are used. A gradient-based adaptive median filter is described in [13]. Segmentation methods [14] group the pixels with similar properties and separate the different pixels. This procedure is based on color, texture, or intensity features. Threshold methods [15] and clustering algorithms are simple and easy to implement. More advanced and complex algorithms, such as morphological [16,17], statistic model-based [18], and graph-based [19] approaches, have also been developed.

Feature detection is an essential element of image processing, enabling reductions of the amount of information necessary for further processing and shortening the computation time. Information about the edges of analyzed objects is critical for SAR imagery. Most canonical approaches to edge detection use Sobel operators [20,21], Laplacians [21], or the Canny edge detector [21]. The Canny edge detector is a much more resource-demanding algorithm. It uses filters and gradient detection to detect preliminary edges. Optimal contours (of single-pixel width) are retrieved based on these edges. Recently, due to the fast development of OpenCV, the algorithm introduced by Suzuki, Abe [22] gained popularity. It applies a hierarchical relationship between the border points, also allowing differentiations of the outer edge from inner object edges.

There are many common algorithms for line detection in a binary image. Most popular are the Hough transform [23] and the fast line detectors (or line segment detectors [24]). An example of a fast line detector (used in OpenCV) is described by Lee et al. [25]. It is much faster than a Hough transform while still providing sub-pixel resolution.

The features strongly depend on the objects (areas) to be detected. For example, L-shaped bright lines can be selected as the features corresponding to buildings [26,27]. Bright rectangular areas typically represent cars, but can represent other vehicles or ground-level metal structures. In [28], an active contour approach has been presented. This method can be used to extract shadows of a building, which can be used to estimate building dimensions. Efficient line- and contour-detection algorithms are described by Suzuki, Abe [22] and Lee [25].

Many different kinds of classification algorithms have been developed. Among them, the nearest neighboring method, naive Bayesian classifiers, SVMs, and neural networks [29] are widely used.

Algorithms vary in accuracy, learning, and answering time. Pre-processing and object detection are time-consuming for high-resolution, noisy SAR images. One can notice that, thanks to the ”visual attention” mechanism, a human can find the objects of interest in complex scenes quickly [30]. Introducing the visual attention mechanism into image processing reduces computational complexity and improves efficiency. To implement a human-like visual attention method, salient regions that often represent meaningful objects must be extracted. Such regions can be recognized based on pixel intensity (visual features).

In recent years, convolutional networks have become very popular. They enable the simultaneous classification and detection of the most critical image features [31]. For example, YOLO (you only look once) deep neural networks dominated the detector-class neural network rankings. At the current moment, there are five versions of YOLO networks. As mentioned in the article by Srivastava et al. [32], YOLOv3 allows for rapid calculation while maintaining very high accuracy. The transition from DarkNet to the PyTorch with the fifth version allowed for the rapid development of YOLOv5 [33], which started with lower accuracy. Due to its current high accuracy and low inference time, YOLOv5 is currently an industrial standard for object detection. For higher processing-power units (or slower applications), region-based CNN (R-CNN) [34], fast R-CNN [35], faster R-CNN [36], or single-shot detector (SSD) [37] can also be used. All of these networks are convolutional neural networks. Additionally, an alternative, transformer neural networks, recently gained popularity in image-processing tasks (for example, the application presented by Carion et al. [38]).

## 3. A Classic Image Processing System Using Contextual Information

This section contains no new (from an image processing point of view) algorithms. We have concentrated on choosing such methods to minimize computation time. We have shown that replacing the classically used Hough transform with LSD improves the classification results. Our approach has been inspired by Zhao et al. [26] and Zhang et al. [27]. In [26], it is shown that the combination of the characteristics (bright lines) of buildings and contextual information (shadows) can overcome the problems of false detection. In [27], the Hough transform is proposed for L-shape detection.

The Hough transform was used for line detection, but during tests, the method had the following disadvantages: it had several tuning parameters and was time-consuming. The results of using the Hough transform were unsatisfactory. The method detected segments that did not exist in the image. We turned to use the fast line setector (FLD) method [27]. FLD is designed for line detection, works without parameter tuning, and has linear computational complexity. The comparison between the Hough transform and FLD is presented in Section 6.2.

L-shape detection, applied to the whole image, can be time-consuming, so we propose the top-down context model [30] visual attention algorithm to accelerate the process. The area of interest is chosen based on contextual information (the shadows area). First, the shadows are detected (contextual areas), and then the adjacent regions are analyzed. As a result, only small parts of the image are analyzed. Significant attention is paid to choosing effective low-level algorithms, such as filtration and edge detection.

The algorithm of object detection consists of the following steps:

- image pre-processing: filtering;
- segmentation: top-down, color-based visual attention [30];
- feature extraction: salient region analysis;
- classification: simple rule-based system.

### 3.1. Image Pre-Processing

Image pre-processing is an essential and time-consuming part of the object-recognition algorithm. It involves removing noise and removing reflections. In order to remove noise, smoothing filters are used. Two types of filters have been tested: a median filter and a bilateral filter. A median filter can reduce impulse noise (salt-and-pepper noise). It requires sorting the values of intensity, so for large-neighborhood matrices, this process becomes time-consuming. A bilateral filter is defined as a weighted average of pixels, and it takes into account the variation of intensities to preserve edges.

The results of salt-and-pepper noise reduction are better for the median filter, but bilateral filtering is faster. Table 1 and Table 2 present the computation time of image filtering.

### 3.2. Segmentation

The purpose of segmentation is to extract salient regions that often represent meaningful objects. This region can be recognized based on pixels’ intensity (color—key features). In our approach, two kinds of salient regions are extracted: shadow regions (black pixels) and bright areas.

**Figure 3:**
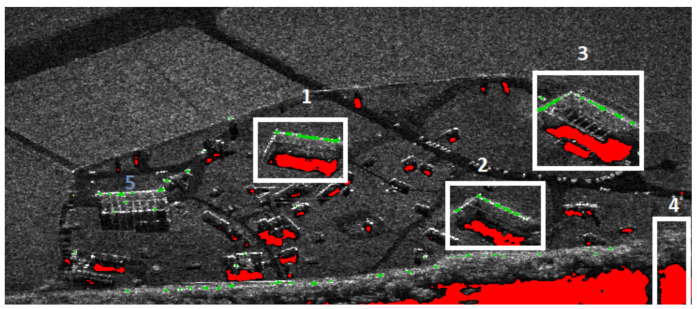
Extracted parts of the image of an urban area.

**Figure 4:**
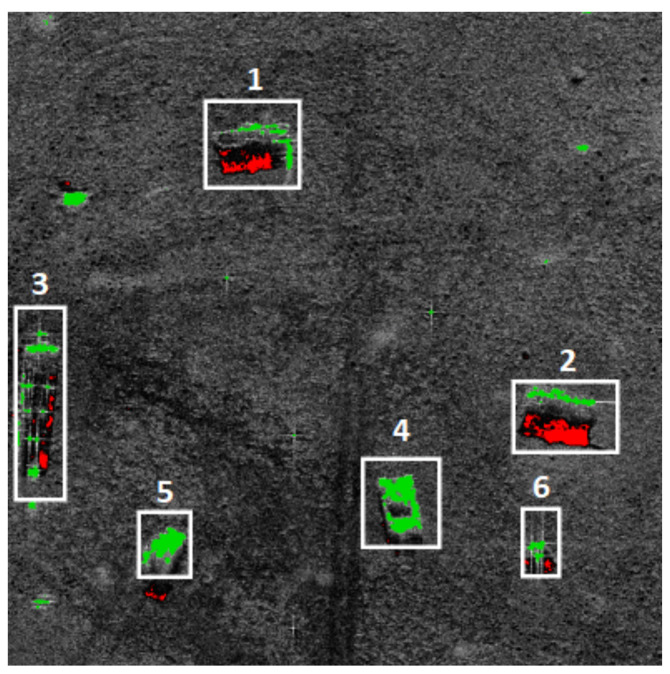
Extracted part of the image of the group of tanks.

Shadow regions on a SAR image are relatively easy to recognize. They can be characterized by an approximately zero illumination level (black area) and the absence of speckle-noise that affects other images’ parts. Figure 3 and Figure 4 present the shadow extraction results in different areas: an urban area, a desert, and a military training ground. In the first image (Figure 3), one can notice that the shadow areas (red pixels) are next to the images of buildings and trees.

Usually, shadows are adjacent to the underlying objects (buildings, trees, etc.), so shadows provide contextual information. The position of the shadow in relation to the object depends on the SAR position relative to the ground. Thus, knowing the location and size of the shadow, one can approximately determine the area in which the object (building) itself is located.

Figure 3 and Figure 4 present the areas selected for further analysis based on shadows. In the first example (Figure 3), the algorithm aims at finding the buildings. It is assumed that the shadows’ area is within 40 × 25 m.

Green pixels represent the brightest areas of the images. The groups of bright pixels are extracted from the image (using thresholding) for further analysis.

### 3.3. Segmented Region Analysis and Classification

Image analysis can be sped-up using a top-down visual attention model [30]. The area of interest is chosen based on contextual information—shadow regions. First, the shadows are detected (contextual areas), and then the adjacent regions are analyzed. The analysis depends on the kind of objects one wants to recognize. In our experiments, two kinds of objects have been identified: buildings and vehicles.

In the case of building recognition, we focus on detecting the L-shaped lines (in an extracted sub-image), which are typical for buildings. Natural objects, such as trees, do not yield long lines in SAR scans.

In Figure 3, four areas (1–4) have been extracted. In areas 1, 2, and 3, L-shaped lines have been found, and those areas are classified as buildings. Area 4 remains unclassified. The algorithm did not detect the hangar (5) because no shadow was detected in its vicinity.

In the second example, the task is to find vehicles (Figure 4). It is assumed that the shadows’ area is within 7 × 3 m. The expected sizes of target surfaces are determined based on the pixel resolution in the SAR scan. Areas 1 and 2 have been recognized as vehicles.

For small or low-height-gradient objects, shadows are not visible. Vehicles (both civilian and military) are among the most popular objects of that type. Due to their high reflectivity, therefore, they can be detected by finding near-rectangular areas occupied by bright pixels. For the cluster of bright pixels, the area of a minimal rectangle is computed to filter noise and define the predicted size of the detected object. In Figure 4, areas 4 and 5 have been classified as vehicles, while areas 3 and 6 have not been classified.

Thanks to the detection of contextual dependencies, the classification error of isolated buildings is minimized. In the case of dense settlements, the method will only detect border (shadow-generating) buildings. Vehicles may be confused with other objects of similar dimensions, e.g., containers. The classification score can be improved by analyzing the image sequence. Vehicles usually change their position, and one can use this information in our analysis. By using object-detection algorithms on SAR scans, which were performed in real-time onboard the UAV, it is possible to reduce the size of the scan sent from the UAV to the ground control station.

## 4. Object Recognition Using the Convolutional Neural Network YOLOv5

We presented an object-detection algorithm using classic machine-vision algorithms in the previous sections. The methods described were to extract parts of the image that may contain objects such as vehicles or buildings. Our next steps were to use the deep learning network YOLOv5 [39]. YOLOv5 belongs to the YOLO family. YOLO (version 1) was introduced in May 2016 by Joseph Redmon. Object detection is defined as a regression problem to spatially separated bounding boxes and associated class probabilities. A single neural network directly predicts bounding boxes and class probabilities from full images in one evaluation. The network is optimized end-to-end directly on detection performance.

YOLO-based object detection is high-speed and processes images in real time.

Redmon et al. [40] introduced YOLOv2. Batch normalization has been added to all the convolutional layers in this network, as has the k-means clustering method to cluster bounding boxes. YOLOv3 improves the detection accuracy and speed but was worse than the previous versions in the case of classifying medium and large objects. Bochkovskiy et al. [41] proposed YOLOv4. YOLOv5 was introduced in 2020.

**Figure 5:**
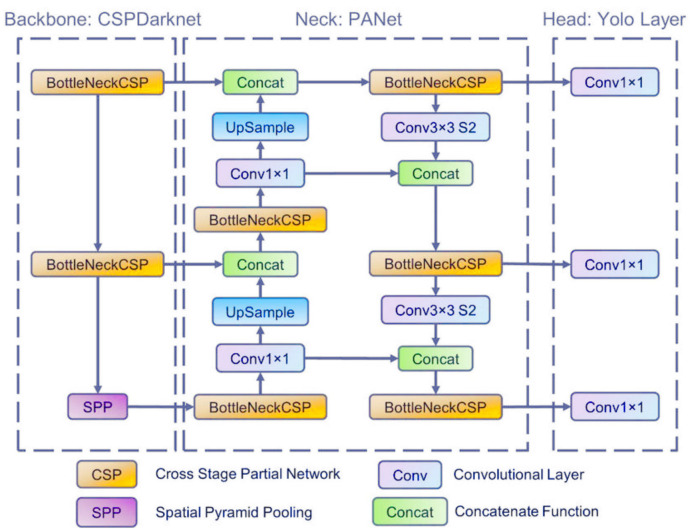
YOLOv5 architecture [44].

Figure 5 presents the architecture of the network. YOLOv5 is a single-stage object detector and consists of three parts: a backbone, a neck, and a head. The backbone is a convolutional neural network that extracts image features from the input image. The neck is the part that contains feature pyramids that help detect hidden features and help to identify the same objects with different scales or sizes. The head part takes features from the neck and makes boxes and class predictions.

**Figure 6:**
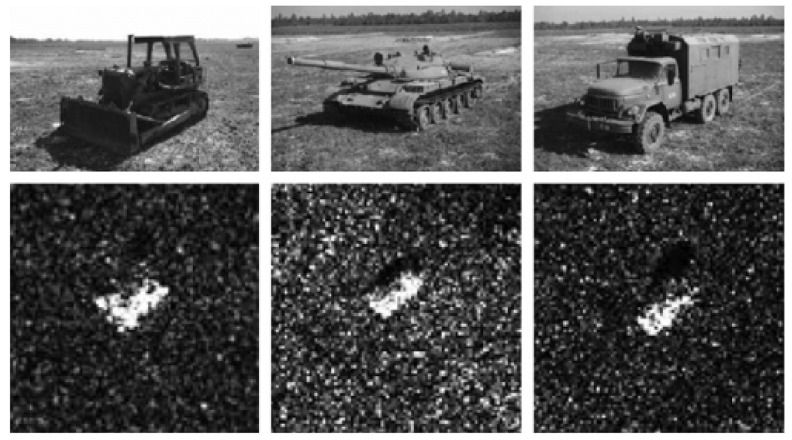
Camera images and corresponding SAR images from MSTAR database [42].

Our experiments combined the MSTAR [42] and SSDD [43] datasets. There are four kinds of objects in MSTAR dataset: 3 kinds of military vehicles and a military building. We combined these three kinds of military vehicles into one type of object and left the military building object as it was. We chose the number of images with ships from the SSDD dataset to match nearly the number of other classes’ images. We manually labeled the images and used the YOLOv5 algorithm to detect and classify the images’ objects. Sample images are presented in Figure 6.

Using the YOLOv5 algorithm, we made detection on unprocessed images of three classes: tanks, ships, and military buildings.

In YOLO networks, three RGB components are provided for each image pixel. The images provided by SARs are gray-scale images, and for each pixel, the values of the three components are duplicated. Our goal was to use context to improve classification accuracy. We compared the mAP (mean average precision) metric to decide whether the detection was better or worse.

We tested three methods of teaching the network, and only objects were selected: objects including their shadows, with the brightest pixels of objects, and with their shadows added to other image channels.

**Figure 7:**
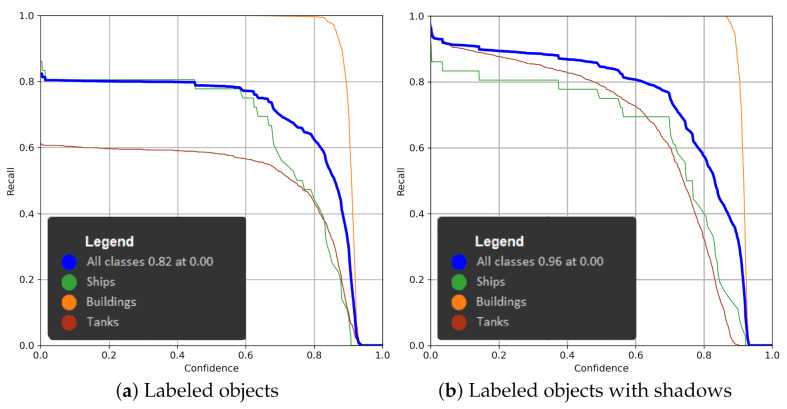
R curve for networks with different preprocessing setups.

**Figure 8:**
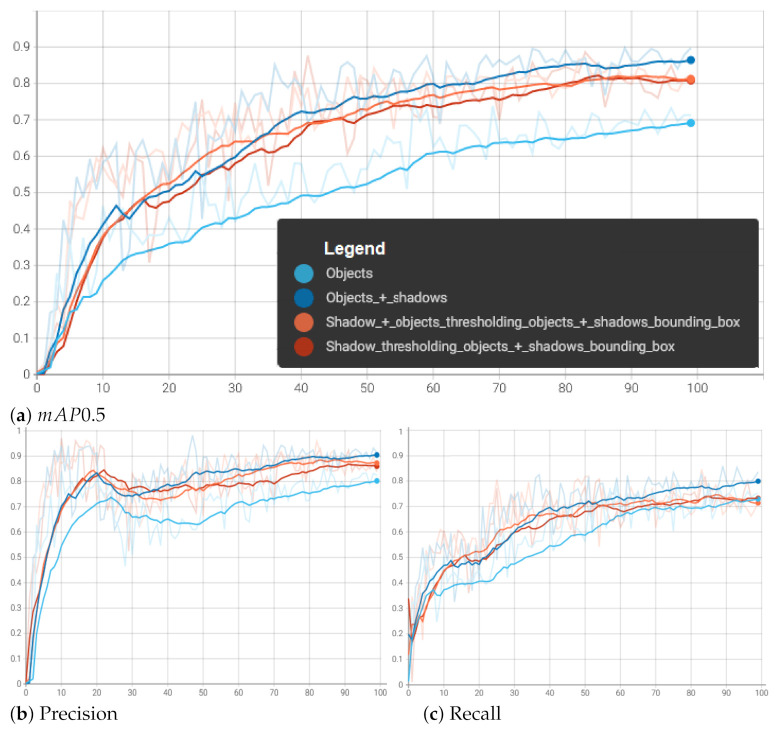
Smoothed metrics’ courses during training for validation dataset of 4 described configurations, featuring mean average precision, precision, and recall—defined in Equations (1)–(3).

As can be seen, the YOLOv5 model is learning quite fast and convergently on this dataset. The detection time is around 20 ms, so that the network can detect objects in real time. The whole R curve is presented in Figure 7. The classification precision equals up to 87% (Figure 8).

Learning curves with three metrics are presented in Figure 8, marked with a light blue color. The metrics are defined in Equations (1)–(3):

(1) $$\mathrm{precision}=\frac{TP}{TP+FP},$$

(2) $$\mathrm{recall}=\frac{TP}{TP+FN},$$

(3) $$mAP=\frac{1}{n}\sum_{i=1}^{i=n}AP_{i}=\frac{1}{n}\sum_{i=1}^{i=n}\sum_{j=1}^{j=k-1}\left(\mathrm{recall}\left(j\right)-\mathrm{recall}\left(j+1\right)\right)\cdot \mathrm{precision}\left(j\right),$$

where TP —true positive, FP —false positive, FN —false negative, *n*—number of classes, $AP_{i}$ —average precision of class *i*, *k*—number of threshold for averaging (between 0 and 1), and recall (x) /precision (x) corresponds to the recall/precision with confidence level of *x* (value of the network’s certainty that this area contains an *i*-class object).

## 5. Improving CNN Using Classical Image Processing

The edges of the boxes generated by the YOLOv5 network are parallel to the edges of the image, so determining the object’s position is very imprecise.

**Figure 9:**
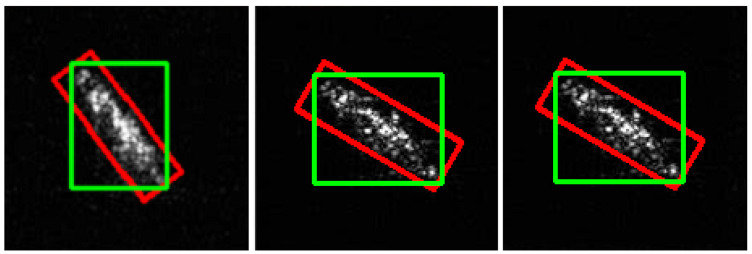
Bounding boxes: green rectangles represent the bounding box generated by YOLO; the red one represents the area marked by classical image-processing methods.

We have used classical image-processing methods (described in Section 3) to determine the minimum area-rotated bounding box. Figure 9 shows the result of the experiments. The bounding box generated by YOLOv5 is shown in green; our algorithm determined the bounding box in red.

**Figure 10:**
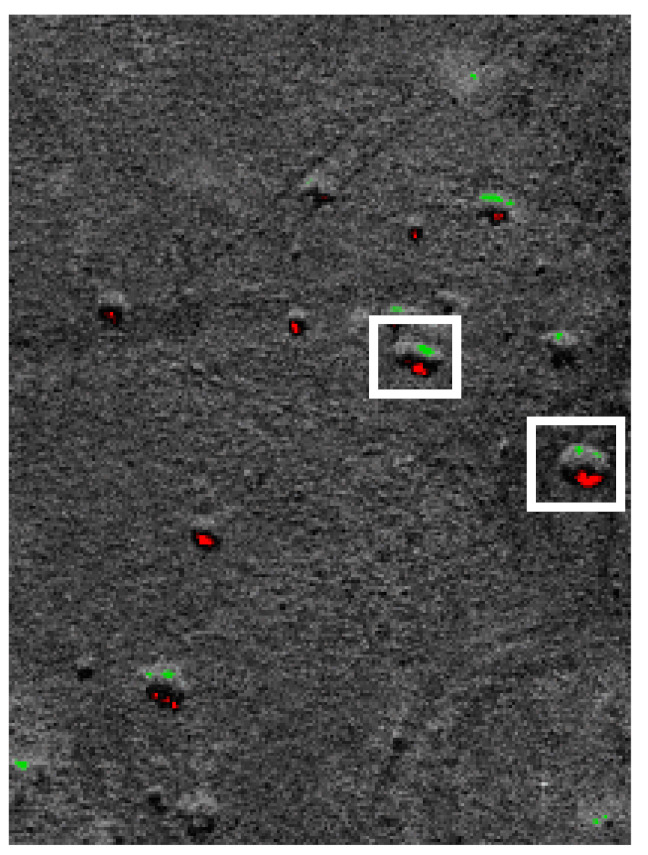
The image of the desert: stones recognized as tanks.

Another problem we face is the relatively high number of false positive classifications. In 20% of the cases, background elements (stones, trees, etc.) were recognized as ships or tanks. Figure 10 shows an image obtained from a SAR in the desert. Rocks in the image have been classified as vehicles.

CNN classifies objects based on their convolutional features, so in the case of similar-looking but different-sized objects, correct classification is not possible without providing metric information. We can specify an acceptable range of parameters for most objects, such as length or width. This information is very useful in the classification process.

As mentioned in Section 1, SAR images are usually accompanied by scan metadata. Based on the metadata, we can determine pixel resolution.

Bounding boxes generated by YOLO are usually too large to determine the dimensions of an object from them. However, the minimum-area bounding boxes created by our method determine the dimensions of objects quite well. By comparing the calculated object dimensions (length and width) with acceptable values for the object type, we can reject the false hypothesis. As the area to be analyzed is small, the analysis time is short (a few milliseconds).

**Figure 11:**
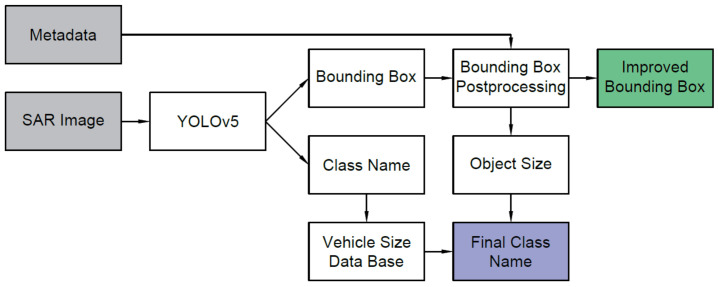
The algorithm’s data-flow graph.

**Figure 12:**
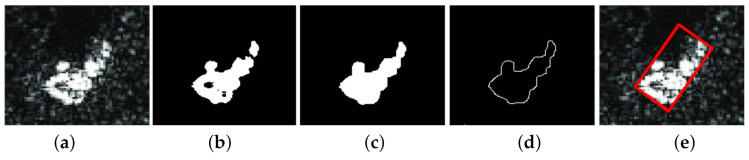
The stages of post-processing: (**a**) bounding box of object detected by YOLO network; (**b**) the result of Otsu segmentation; (**c**) the result of morphological closing; (**d**) the result of applying Canny edge detector; (**e**) minimal area bounding box.

Figure 11 shows the diagram of the proposed classification algorithm. In summary, the algorithm’s steps are as follows:

- We train the network;
- The SAR image is the input of the YOLOv5 network;
- In the network’s output we obtain a list of names of recognized classes and their corresponding bounding boxes;
- The area inside the bounding box is analyzed, the dimensions of the detected object are determined, and a new bounding box with the minimum area is determined. Figure 12 shows the stages of bounding box post-processing;
- The calculated value of the object parameters is compared with the range of permissible values for the given object class, and the hypothesis provided by YOLOv5 is confirmed or denied.

## 6. Experimental Results

This article deals with SARs that can be mounted on platforms with a maximum take-off weight (MTOW) of up to 150 kg (in military nomenclature, these are tactical-class systems). An example of this type of system is the ARS-800 SAR [45] or Leonardo’s PicoSAR [1]. The capabilities of the radars in terms of reconnaissance depend strictly on how they are mounted on the platform and the platform’s capabilities.

Popularly used in UAVs, the tested computing unit is a Jetson Tegra TX2i System on a Chip (SoC)-class computer from Nvidia. Its main advantages are low power consumption, high computing power, robustness, an industrial standard, and a separate specialized Nvidia Pascal™ architecture GPU with 256 Nvidia CUDA cores.

The following tests were conducted with all primary power settings, as Jetson TX2i supports a few processor power configurations. For the sake of brevity of description, only two power modes will be discussed:

- MAXQ—maximizing power efficiency—power budget up to 10 W, 1200 MHz Cortex A57 CPU, 850 MHz built-in Nvidia Pascal GPU with 256 CUDA cores,;
- MAXN—maximizing processing power—power budget around 20W, 2000 MHz Cortex A57 CPU, 2000 MHz Denver D15 CPU (not utilized in our tests), and 1300 MHz built-in GPU.

All tests were conducted on Linux Ubuntu 18.04 with the newest Nvidia SDK and CUDA driver (Cudart 10.2).

In computationally demanding operations with parallel tasks, GPUs take advantage of their architecture to achieve higher speeds of calculations and lower power consumption. The main limitation of using the GPU is the need for data uploads to and from the GPU’s VRAM (memory), which is time-consuming. Thus, they reach maximum efficiency when data is heavily processed.

In the case of embedded systems, where real-time operations are required, an additional advantage of using GPU is using the full CPU capabilities during the image processing simultaneously. It is also possible to transfer all image data directly to the GPU (without the CPU and its bus lanes). Some devices support RDMA (remote direct memory access), allowing external devices to access RAM or VRAM directly. Jetson allows for bypassing the CPU while reading the CSI input to the GPU. Finally, the previously mentioned advantage of low power consumption on the GPU (especially CUDA cores themselves) is also significant.

For comparison, computation times on a personal computer are juxtaposed. The PC used: Intel Xeon E-2176G (12) @ 4.700GHz (Intel, Santa Clara, CA, USA), Nvidia GeForce GTX 1050 Ti (with 768 CUDA cores, 1392 MHz)(Nvidia, Santa Clara, CA, USA), Debian GNU/Linux 10 (buster) x86_64 Operating system, OpenCV 4.2.0, libcudart12.2.

All algorithms are written and executed in C++ 14 standard using a GCC compiler.

### 6.1. Shadow Detection—Processing Time

The results for a series of SAR images pre-processed with a median filter are shown in Table 1. The CPU-based calculation is faster in each case. Further analysis has shown that a significant part of the whole computing time is used for filtering. A median filter requires sorting, which is not optimized for GPU operations.

For better parallelization, the experiment was conducted using a bilateral filter. The average measured times for a series of 100 scans are shown in Table 2. In this case, the difference between CPU and CUDA calculations is much smaller. The GPU calculations’ times contain the image download to RAM because the last steps of the algorithm require a general-purpose CPU.

Due to time-limited resources, the segmentation and sub-images’ generation are conducted before advanced post-processing. This dramatically reduces the size of the analyzed scan fragments with low-demanding operations before applying more advanced operations.

It is worth noting that in the case of Jetson Tegra, the solution using CUDA-supported functions is the most efficient resource-wise and regarding power consumption. In the case of a different GPU architectures, OpenCL counterparts should be used to minimize the CPU load.

### 6.2. Classification—Processing Time

The first step in our classification algorithm is to perform line detection. The Hough transform and fast line detection (FLD) are compared in Table 3.

**Figure 13:**
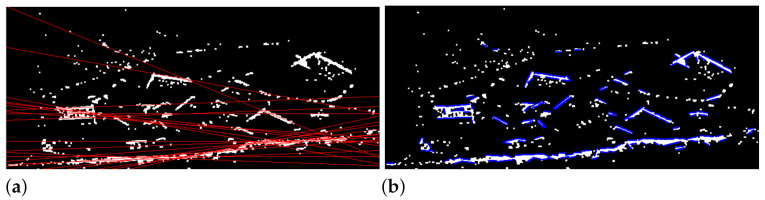
The lines found in the sub-image—comparison of the Hough algorithm (**a**) versus the fast line detector (**b**).

Example results of line detection algorithms of another scan are shown in Figure 13. If a line is found within a sub-scan of a given area, it is assumed that there is an object in the analyzed area that should be recognized. The portion of the image where the line was found should be cut from the scan and transmitted to the ground control station for further analysis. Table 3 presents the processing times of both algorithms. The Hough transform requires the definition of the resolution of the output: $\theta_{res}$ —angle resolution and $\rho_{res}$ —distance resolution (equals 1 pixel in both tests). The Hough algorithm also requires an a priori declaration of expected segment lengths, and as a result, it outputs the equations for the straight line instead of line segment coordinates—which can be observed in Figure 13. It is worth mentioning that the fast line detector does not require these inputs and can interpolate the output values to sub-pixel levels. It does also support gray-scale images, while the Hough transform does not. The results of the algorithms (see Figure 13b) show that the fast line detector algorithm provides correct results for SAR scan analyzes. Hough’s algorithm is unable to extract edges correctly.

In the case of vehicles, the FLD algorithm is less accurate due to the size of the vehicle, the irregularity of the vehicle shape, and the SAR scan’s resolution. For the better detection of straight lines, which indicate the vehicle’s position in the field, sparkling points were used, the presence of which indicates that the vehicle may be in the field.

For the next test, the whole-shadow-detection algorithm with building classification (using FLD) was launched. The results are given in Table 4. The classification algorithm proposed in this paper bears little load to the CPU (around 10% of the presented algorithm’s total computing time). Thus, it was not programmed for native GPU computation, as this would bring minimal profit for CPU load reduction or computing times.

The classification time depends on the size of the sub-images and the number of classified objects. Example times needed for computing sub-images, presented in Figure 3, are listed in Table 5.

For the Hough transform $\theta_{res}$ = 1°, $\rho_{res}=1$ pixel was set. Filters were set up according to the previous description. For brevity, only the CPU processing time was shown in the comparison juxtaposed in Table 6. Using GPU computing would extend the time needed for median filter and, while the Hough transform is easy to calculate in parallel, it has problems with concurrent memory access, significantly limiting its speed with CUDA implementation. As can be seen, the changes proposed to the algorithms effectively limited the resource consumption and noise effect on the output.

In comparison to the final times of our proposed algorithm, the Hough transform requires around 30% more time to perform the analysis, while the median filter adds around 75% to the computational time.

Additionally, the application of visual attention leads to processor load reduction. In the described examples, with only four areas of interest, the time required to conduct FLD with an L-shape detector was around 0.420 ms (for PC, according to Table 5), while the same set of operations for the whole image took about 5.2 ms. The last result is close to the whole processing time for the PC with CPU only (Table 4).

## 7. Conclusions and Future Work

The paper presents a new concept of classification methods: YOLOv5 is used to classify objects and determine the areas in which objects are located. In the next step, classical image-processing methods are used to accurately determine the position of objects and reduce the number of false-positive classifications. Each described algorithm has been implemented on the Jetson Tegra TX2i mobile platform. The capabilities of the computer’s CPUs were compared against the GPU with CUDA. The methods of accelerating image-data transmission from UAV to GCS related to minimizing the analyzed scans were indicated. A lot of attention is brought to choosing efficient low-level algorithms, such as filtration and edge detection. Finally, it is worth emphasizing that the image analysis algorithms presented in the article can be used to determine the speed of the UAV, relative to the ground, to accurately determine the geolocation of the UAV in the absence of a GPS signal. We will use ensemble learning to improve the reliability and accuracy of predictions in future work.

## Figures and Tables

**Table 1:** Pre-processing times, in ms with a median filter and shadow detection.

		CPU	CUDA GPU (with Image Upload)	CUDA GPU (with Uploaded Image)
TX2i	MAXQ MAXN	45.0 26.6	62.3 48.7	54.8 38.3
PC—Xeon 4.7GHz		9.18	12.70	10.15

**Table 2:** Pre-processing times, in ms, with a bilateral filter and shadow detection.

		CPU	CUDA GPU (with Image Upload)	CUDA GPU (with Uploaded Image)
TX2i	MAXQ MAXN	33.3 20.0	39.4 25.8	18.7 15.9
PC—Xeon 4.7GHz		5.76	5.48	2.04

**Table 3:** Time efficiency comparison of the Hough and fast line detection algorithms in OpenCV, in μs.

Algorithm	FLD	Hough $\theta_{\mathrm{res}}$ = 1° $\rho_{\mathrm{res}}=1\mathrm{px}$	Hough $\theta_{\mathrm{res}}$ = 2° $\rho_{\mathrm{res}}=1\mathrm{px}$
Image 300 × 700 px	9418	32,840	10,132
Image 50 × 60 px	113	217	114

**Table 4:** Time of classification, in ms, with shadow recognition using FLD.

		CPU	CUDA GPU (Image Upload)	CUDA GPU with VRAM	YOLOv5	YOLOv5 with Pre-Processing
TX2i	MAXQ MAXN	36.2 21.5	41.8 29.3	27.9 19.7	156 112	156 113
PC—Xeon 4.7 GHz		6.44	6.51	2.98	14.7	14.9

**Table 5:** Subimages’ classification times, in µs.

		No 1	No 2	No 3	No 4
Size		56 × 67 px	46 × 108 px	51 × 135 px	36 × 108 px
TX2i	MAXQ MAXN	118.1 192.4	398.0 646.0	296.2 481.9	265.1 431.8
PC—Xeon 4.7 GHz		47.0	154.5	114.4	101.3

**Table 6:** Final computing times comparison, in ms.

		FLD with Bilateral	Hough with Bilateral	FLD with Median
TX2I	MAXQ	69.1	85.2	108.1
	MAXN	40.8	51.0	65.2
PC—Xeon 4.7 GHz		11.9	16.8	18.9

## Data Availability

Not applicable.
